# Life-Saving Precision: Image-Guided Interventions Transforming Outcomes in Living-Donor Liver Transplant Complications

**DOI:** 10.7759/cureus.81613

**Published:** 2025-04-02

**Authors:** Bribin Bright, Manish M Nair, Srikanth Moorthy, Sreekumar K P., Chinmay Kulkarni, Nazar P K.

**Affiliations:** 1 Radiodiagnosis, Amrita Institute of Medical Science, Kochi, IND

**Keywords:** biliary complications, hepatic venoplasty, image-guided interventions, interventional radiology, living-donor liver transplantation (ldlt), minimally invasive procedures, percutaneous transhepatic biliary drainage (ptbd), postoperative management, transjugular intrahepatic portosystemic shunt (tips), vascular complications

## Abstract

Living-donor liver transplantation (LDLT) is a preferred treatment modality for patients with end-stage liver disease. However, the incidence of postoperative complications, particularly involving the biliary and vascular systems, remains significant. These complications often necessitate urgent interventional management to prevent graft loss. Although surgical revision is an option, it carries increased morbidity and mortality risks. This case series explores the utility of minimally invasive, image-guided techniques for managing complex post-LDLT complications.

We present four cases involving distinct image-guided interventions, including percutaneous transhepatic biliary drainage (PTBD) with balloon cholangioplasty for biliary strictures, hepatic venoplasty with intravascular stenting for hepatic venous outflow tract obstruction, and transjugular intrahepatic portosystemic shunt (TIPS) placement for refractory ascites secondary to portal hypertension. Procedural techniques, immediate outcomes, and follow-up results were assessed.

All interventions were technically successful, with immediate clinical and biochemical improvement observed in each patient. Follow-up imaging confirmed patency and resolution of the vascular or biliary complications. This series underscores the efficacy of image-guided interventions as a safer alternative to surgical re-exploration in complex post-transplant cases.

Image-guided interventions, including PTBD, venoplasty, and TIPS, offer robust management solutions for biliary and vascular complications in LDLT recipients, highlighting the role of interventional radiology in post-transplant care.

## Introduction

Living-donor liver transplantation (LDLT) has significantly broadened access to liver transplantation; however, it is associated with distinct postoperative complications, primarily involving the biliary tree and hepatic vasculature. Biliary complications, such as strictures and leaks, affect approximately 20-30% of LDLT recipients and, if not promptly managed, can result in graft failure [1]. Similarly, vascular complications, including hepatic vein or inferior vena cava (IVC) stenosis, may precipitate hepatic venous outflow obstruction (HVOO) and subsequent graft dysfunction.

While surgical re-intervention remains a treatment option, it is often associated with elevated morbidity and mortality due to anatomical complexity in post-LDLT patients. Conversely, minimally invasive, image-guided procedures such as percutaneous transhepatic biliary drainage (PTBD), balloon venoplasty, and transjugular intrahepatic portosystemic shunt (TIPS) placement have emerged as effective alternatives [2-10]. This case series details the application of these interventional techniques in four complex cases of post-LDLT complications.

## Case presentation

**Figure 1:**
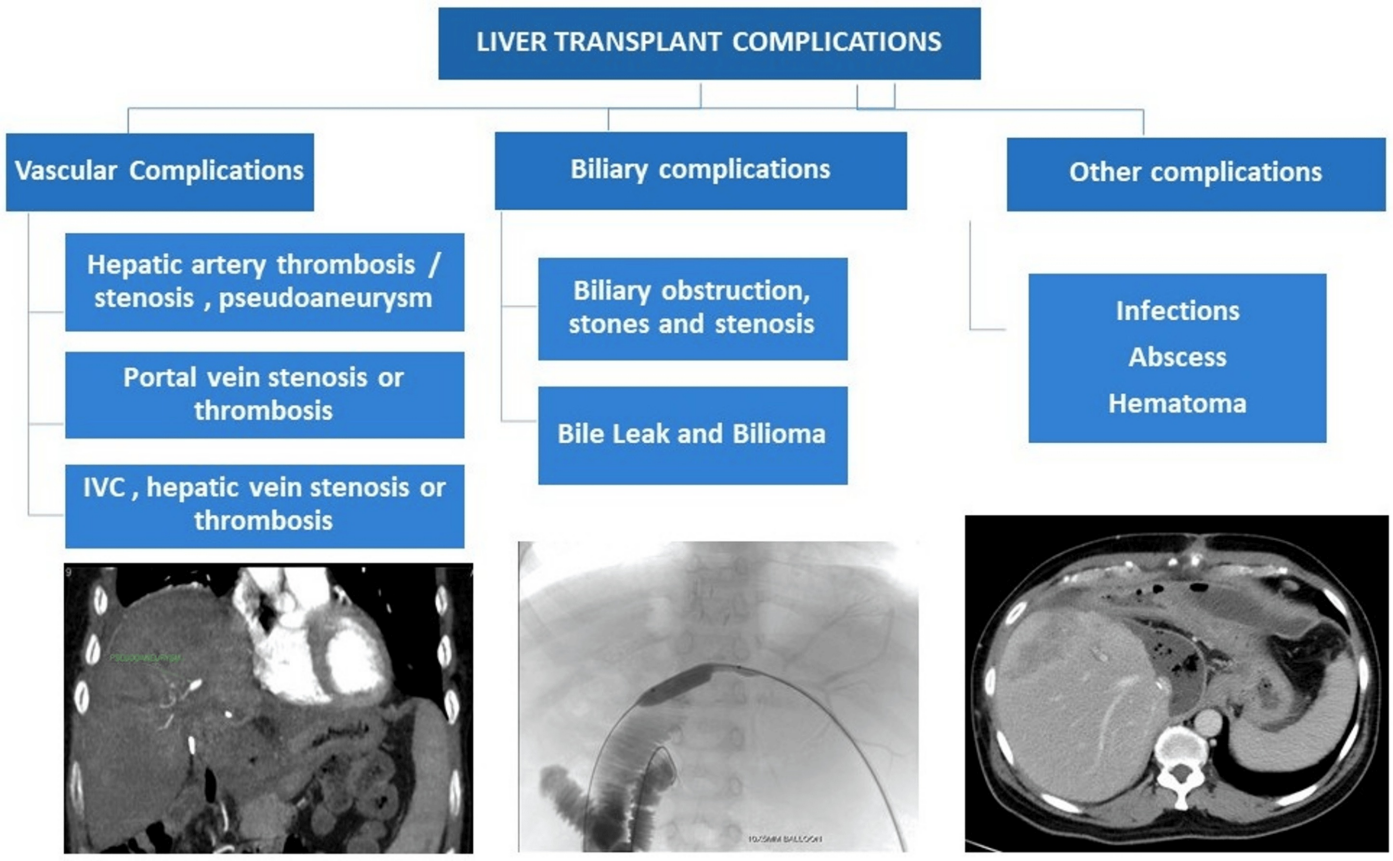
List of post-LDLT complications IVC: Inferior vena cava; LDLT: Living-donor liver transplantation Figure credits: Author Bribin Bright

Before presenting the individual cases, it is important to emphasize the relevance and impact of these image-guided interventions in post-LDLT management. With the increasing complexity of liver transplant cases, prompt and effective treatment of complications is vital to ensure graft survival and patient well-being. Figure 1 enumerates various complications in post-LDLT.

Vascular interventions

**Figure 2:**
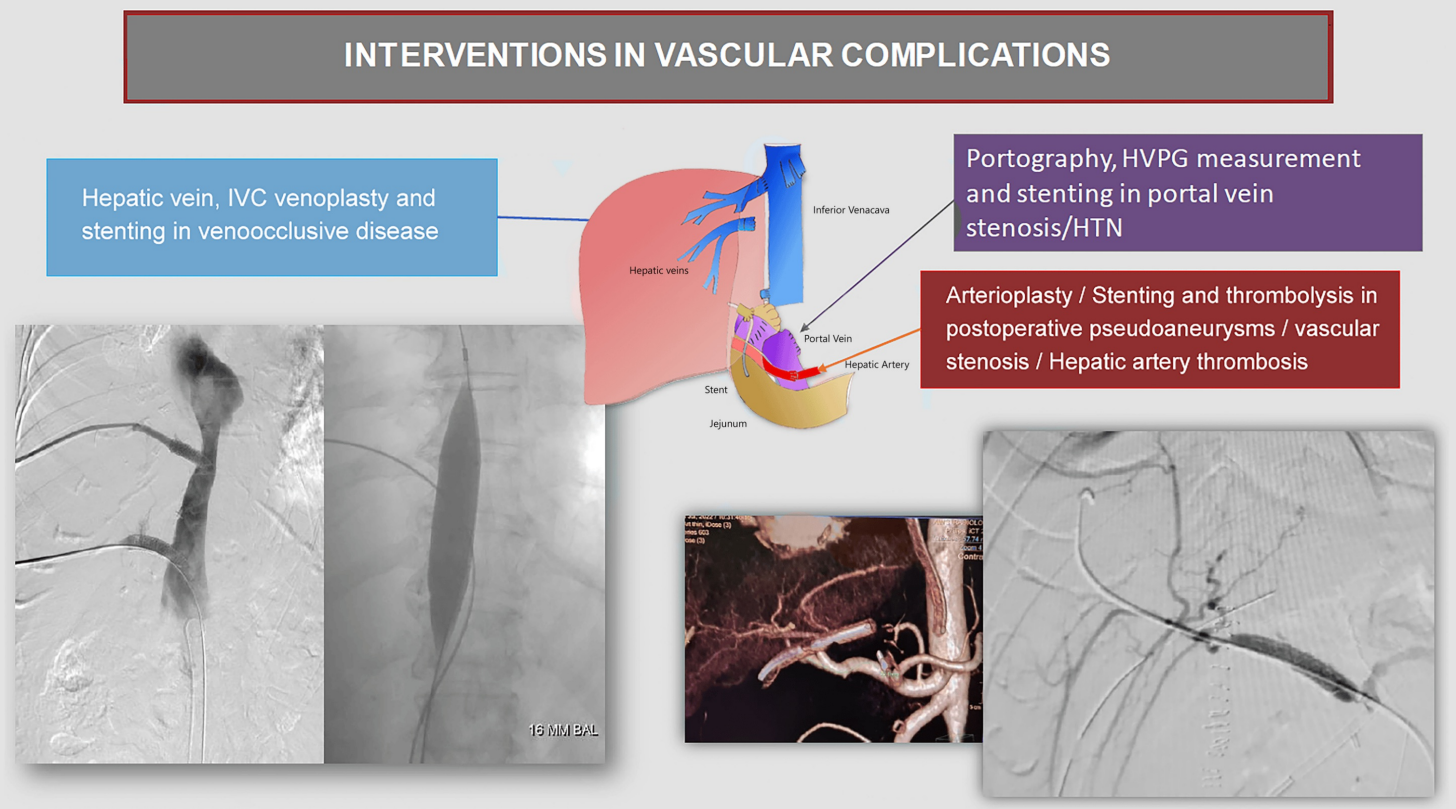
Various minimally invasive treatment options in post-LDLT vascular complications IVC: Inferior vena cava; LDLT: Living-donor liver transplantation; HVPG: Hepatic venous-portal gradient; HTN: Hypertension Figure credits: Author Bribin Bright

Balloon venoplasty with stenting offers a definitive solution for HVOO in LDLT recipients, where altered anatomy and collateral circulation often complicate open surgical intervention (Figure 2). Studies indicate that percutaneous venoplasty and stenting maintain long-term patency in hepatic veins and the IVC, improving hemodynamic parameters and reducing postoperative morbidity.

Biliary interventions

**Figure 3:**
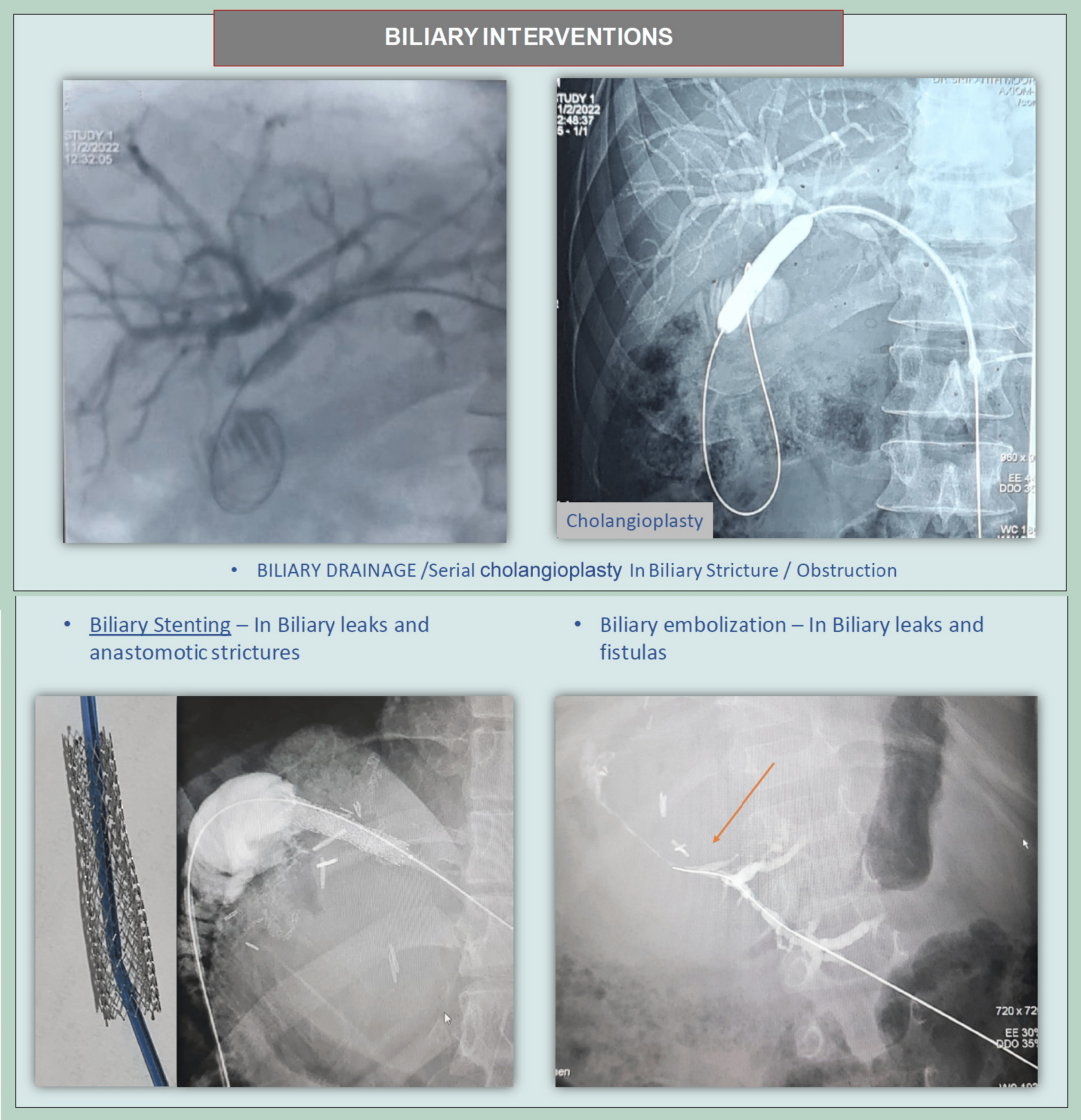
Options in biliary complications

PTBD with balloon cholangioplasty is a recognized approach for managing complex anastomotic biliary strictures (Figure 3). Literature supports PTBD as an effective strategy for stricture management in cases where endoscopic retrograde cholangiopancreatography (ERCP) is unsuccessful. The technique enables direct access to intrahepatic ducts, providing precise dilation of stenotic segments with low complication rates.

Portal hypertension

The TIPS procedure is well-established for the management of portal hypertension and its sequelae, including refractory ascites. TIPS placement in one of our cases alleviated portal pressure, promoting hemodynamic stability and reducing the need for frequent paracentesis. This supports existing evidence that TIPS is a safe and effective option for managing portal hypertension in post-LDLT patients. 

The following case presentations illustrate how minimally invasive procedures, such as PTBD, hepatic venoplasty, and TIPS placement, can provide significant benefits, reducing the need for more invasive surgical re-exploration and improving overall patient outcomes.

Case 1: Management of anastomotic biliary strictures in a pediatric LDLT recipient

Patient History

**Figure 4:**
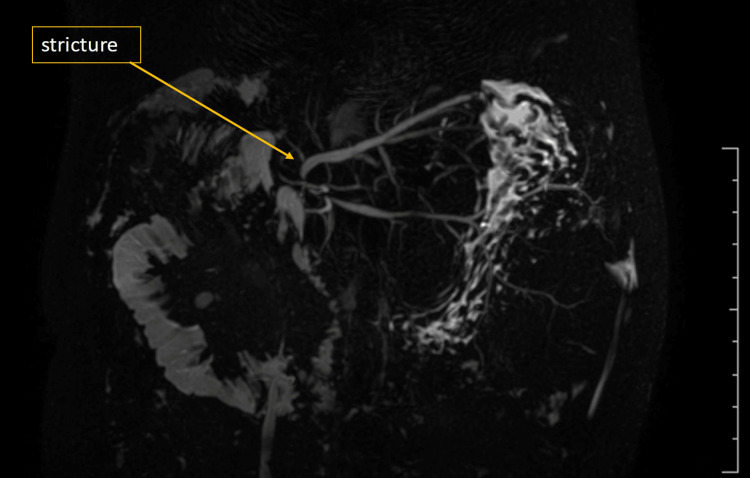
3D MRCP showing biliary anastomotic stricture with hepatic hilar collection MRCP: Magnetic resonance cholangiopancreatography

A three-year-old male with type IIb biliary atresia underwent LDLT. Postoperative imaging at three months revealed a hepatic hilar collection and biliary anastomotic stricture involving segments 2 and 3, confirmed on magnetic resonance cholangiopancreatography (MRCP) (Figure 4).

Intervention

**Figure 5:**
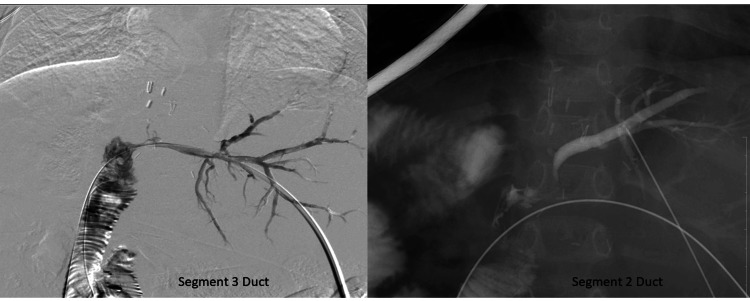
Cholangiogram showing two strictures involving segment 2 and 3 ducts , which were accessed percutaneously using a Chiba needle (Cook Medical, Bloomington, USA) and crossed successfully with a Terumo wire (Terumo Interventional Systems, USA)

**Figure 6:**
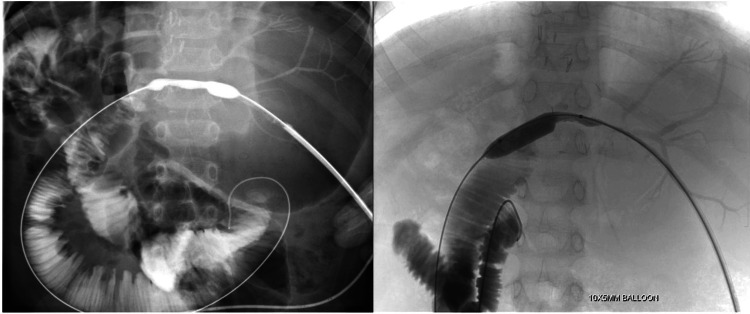
Percutaneous intervention - serial balloon dilation for strictures in segment 2 and 3 ducts

Initial management included failed attempts at ERCP due to the complexity of strictures. Therefore, PTBD was employed, utilizing a percutaneous Chiba needle (Cook Medical, Bloomington, USA) for transhepatic access to the biliary system. Under fluoroscopic guidance, a 0.018-inch microwire (Cook Medical, Bloomington, USA) was advanced through the strictures, followed by sequential balloon cholangioplasty using 4 mm and 8 mm balloons over a period of two months (Figures 5-6). In view of the failed ERCP and benign nature of the stricture, PTBD was done with serial biliary dilatations over a period of two months.

Outcome

**Figure 7:**
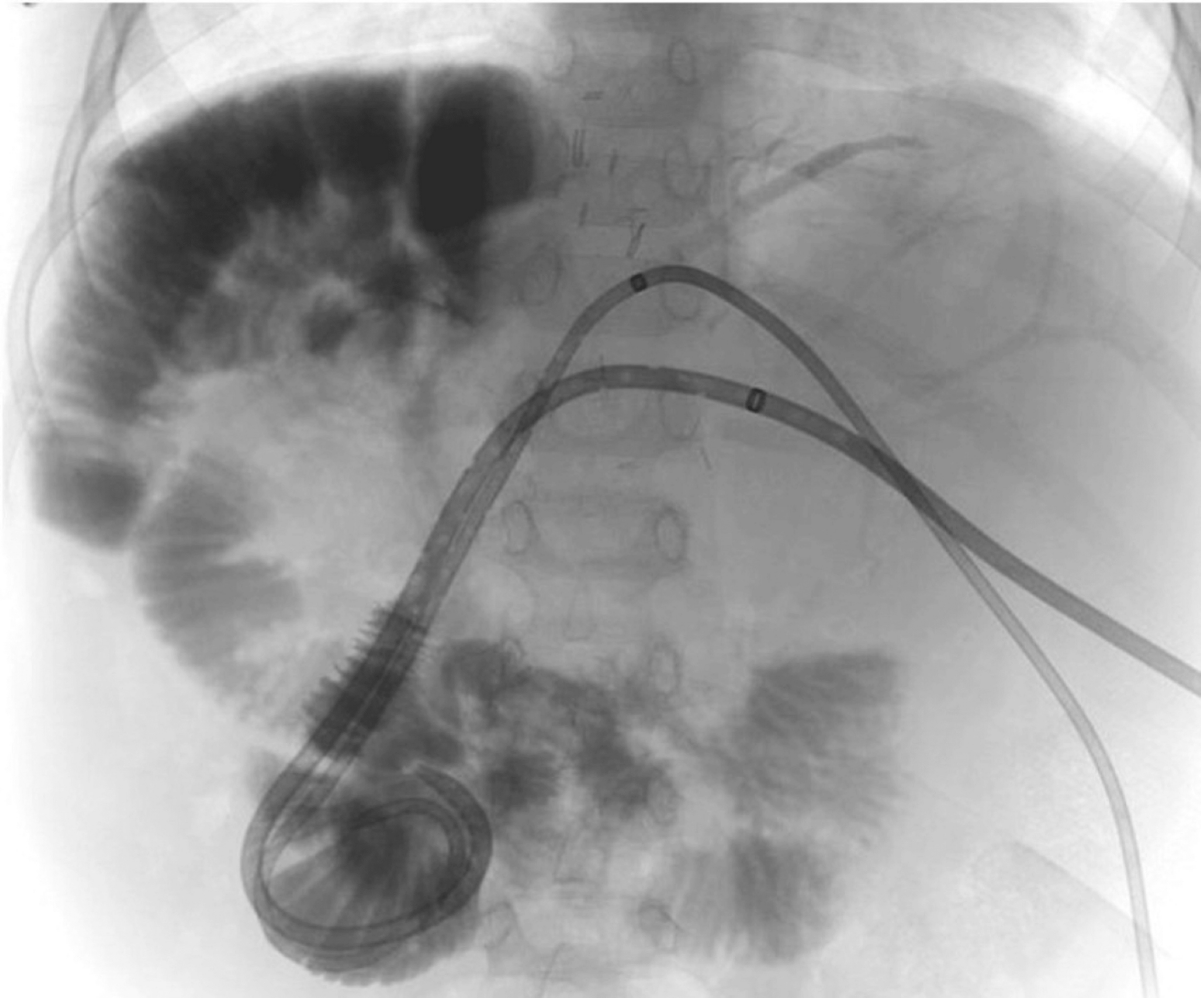
Follow-up cholangiogram showing patent biliary anastomosis with good flow across the reconstructed biliary anastomotic site

Follow-up cholangiography demonstrated good bile flow across the anastomotic site (Figure 7). Liver function tests (LFTs) normalized, and hepatic hilar collections resolved on subsequent ultrasound. This percutaneous approach circumvented the need for repeat surgical intervention and achieved sustained patency in the biliary tree.

Case 2: Hepatic venous outflow obstruction in an adult LDLT recipient

Patient History

**Figure 8:**
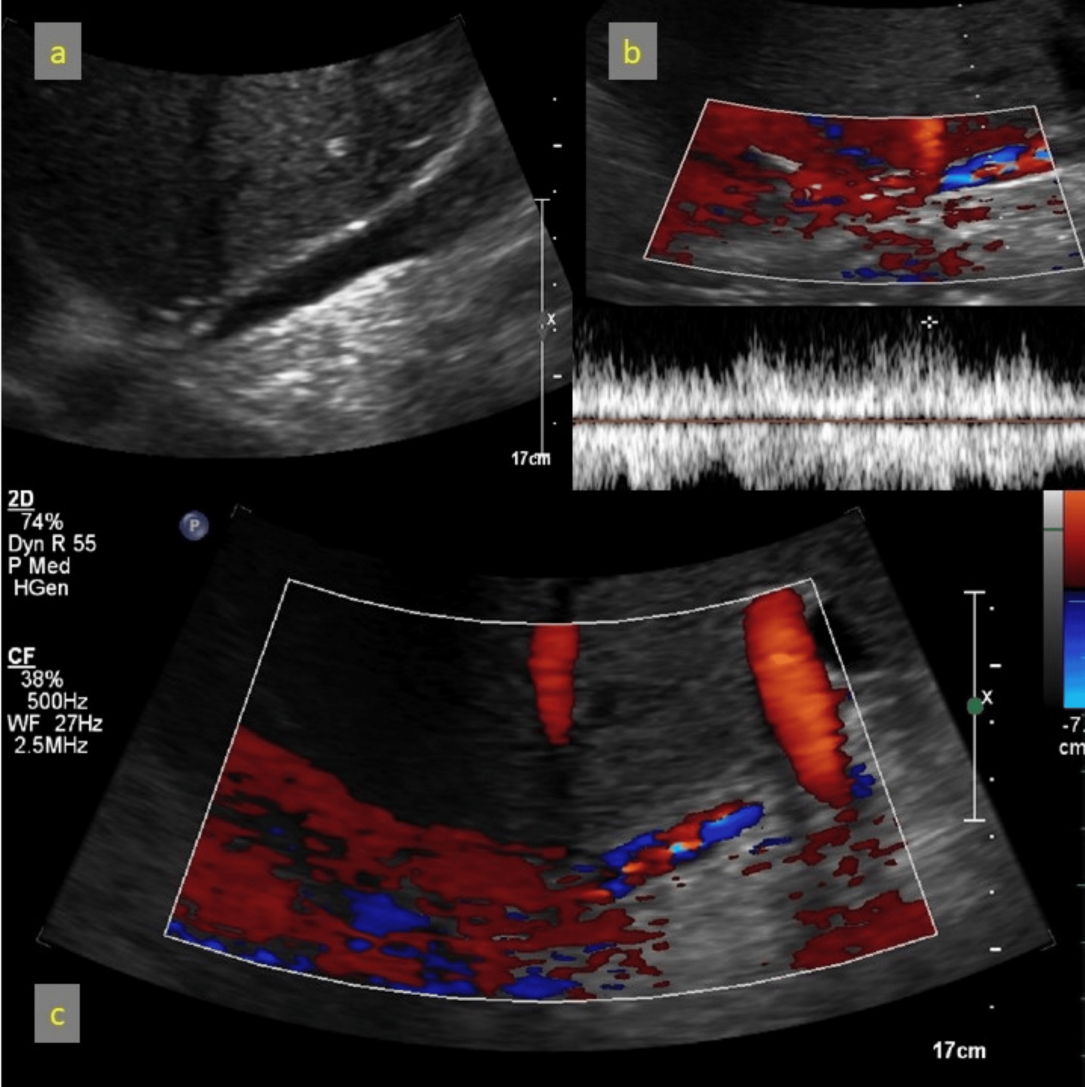
(a) Retrohepatic IVC narrowing on USG; (b) Turbulent flow on Doppler sonography; (c) Reversal of flow in the right and middle hepatic veins on Doppler sonography IVC: Inferior vena cava; USG: Ultrasound

**Figure 9:**
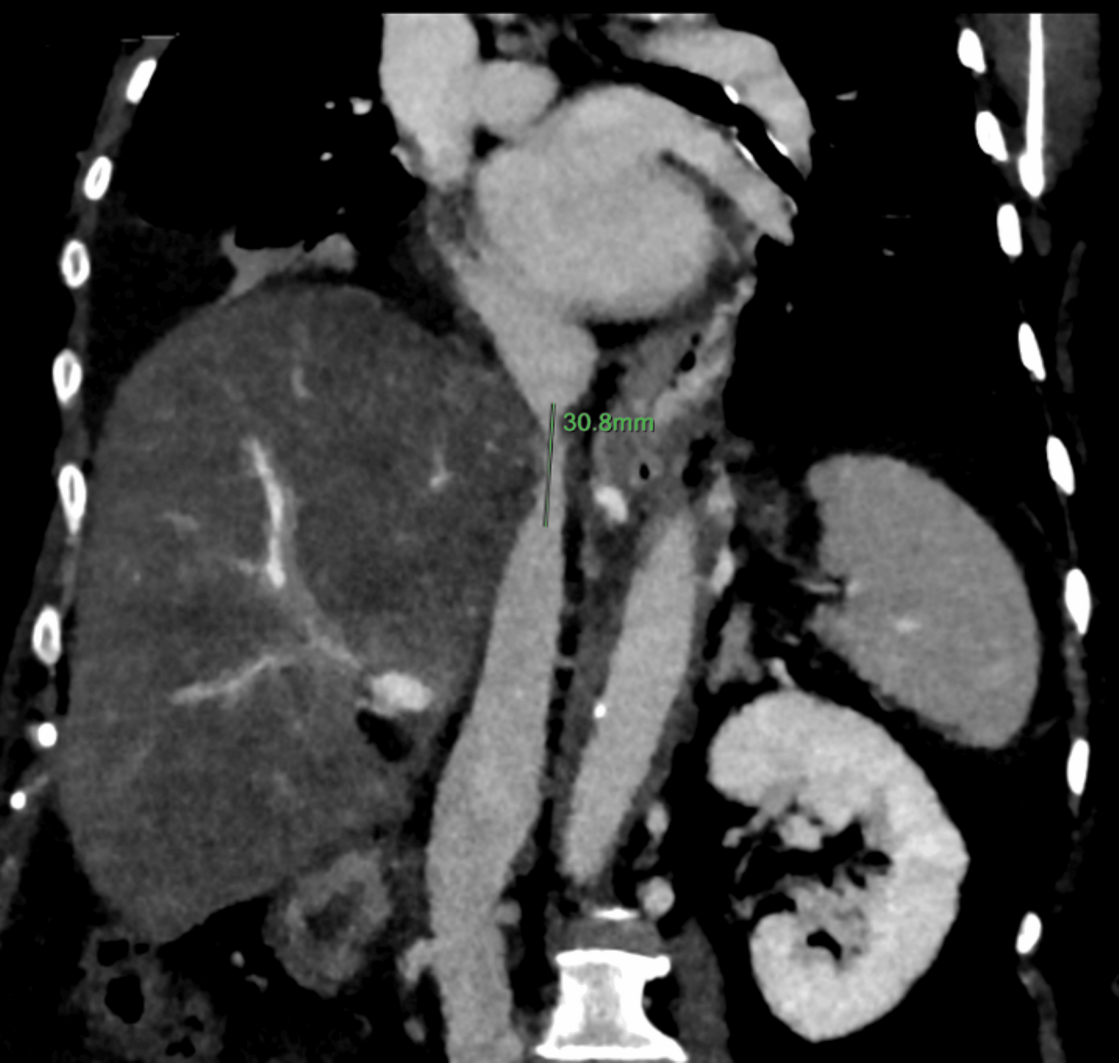
Delayed phase post-contrast coronal CT section showing narrowing of the hepatic and retrohepatic IVC segment with occlusion of the hepatic veins CT: Computed tomography; IVC: Inferior vena cava

A 58-year-old male post-LDLT presented with significant HVOO and stenosis at the hepatic and retrohepatic segment of the IVC and occlusion of the right and reconstructed middle hepatic veins at the ostia. Duplex Doppler sonography demonstrated reversed hepatic venous flow (Figure 8), while computed tomography (CT) imaging revealed retrohepatic IVC narrowing with compensatory collateral venous channels (Figure 9).

Intervention

**Figure 10:**
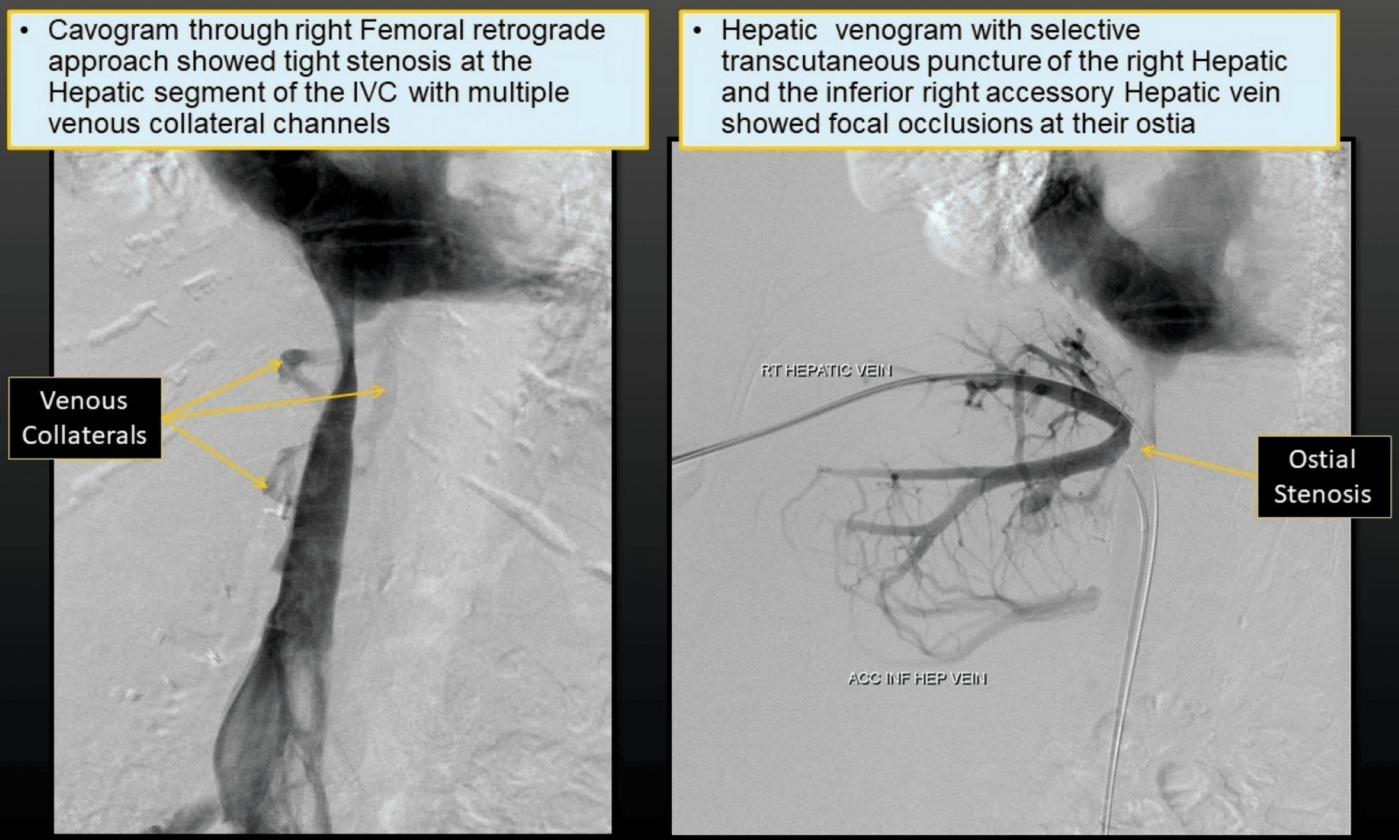
Cavogram showing tight stenosis in the hepatic IVC segment with venous collaterals and focal occlusions in the hepatic vein IVC: Inferior vena cava

Venography via a femoral retrograde approach confirmed the IVC stenosis (Figure 10). The stenotic segment was dilated using a 16 mm x 4 cm high-pressure balloon. In addition, a balloon-expandable intravascular stent (Visi-Pro Peripheral Stent System Medtronic, Minneapolis, USA) was deployed across the occluded hepatic veins to restore patency.

Outcome

**Figure 11:**
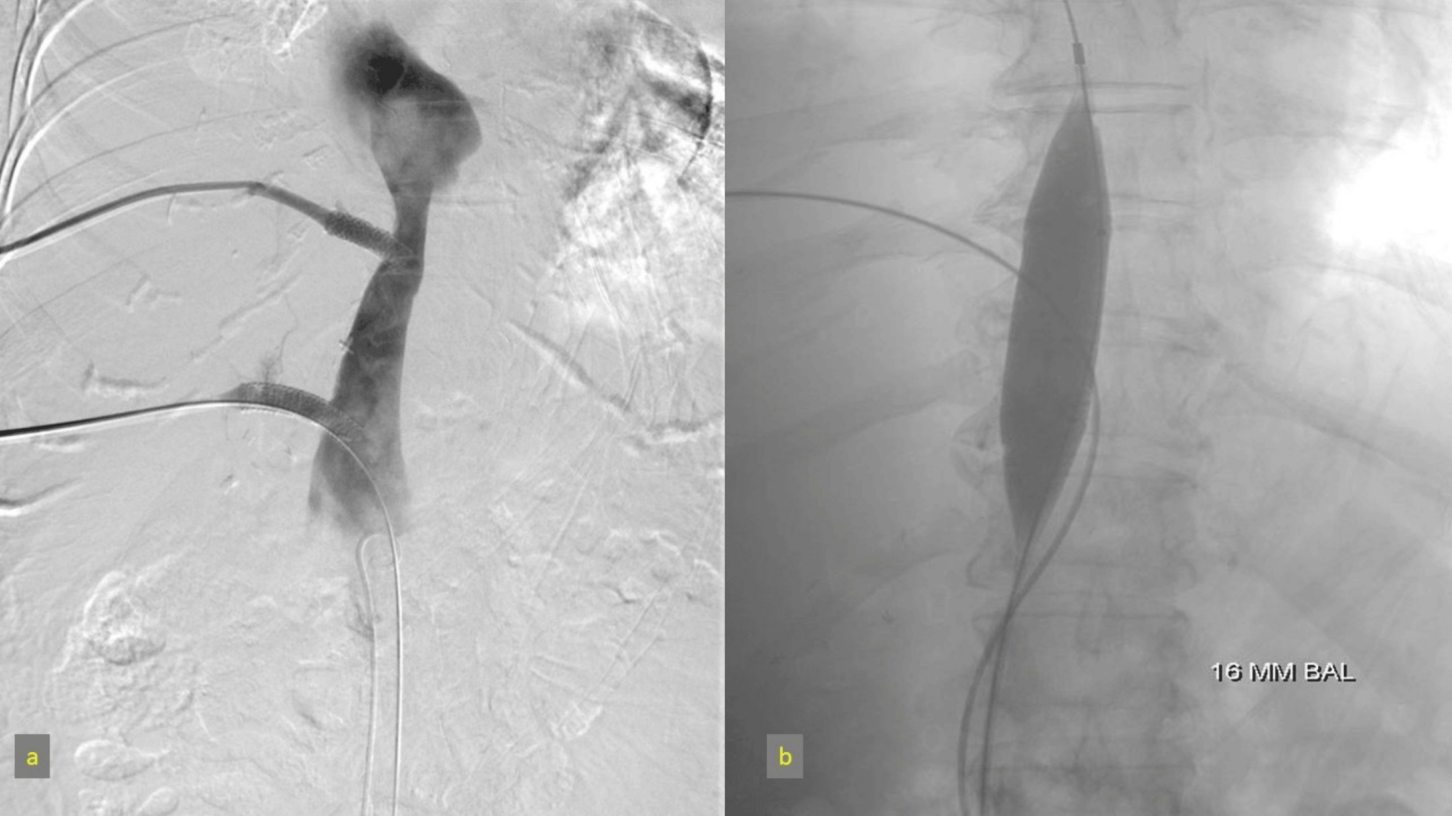
IVC stenosis balloon angioplasty and hepatic vein stenting IVC: Inferior vena cava

Post-venoplasty and stent placement, follow-up venography confirmed resolution of the outflow obstruction, with normalized flow in the hepatic veins and IVC (Figure 11). This intervention stabilized the patient’s hemodynamics, relieved ascites, and restored liver function, thus proving effective in addressing HVOO without surgical risks.

Case 3: TIPS for refractory ascites and portal hypertension

Patient History

A 52-year-old male post-LDLT exhibited refractory ascites and signs of portal hypertension unresponsive to medical management, necessitating repeated paracentesis. Doppler ultrasound and CT imaging revealed portal hypertension secondary to graft congestion.

Intervention

**Figure 12:**
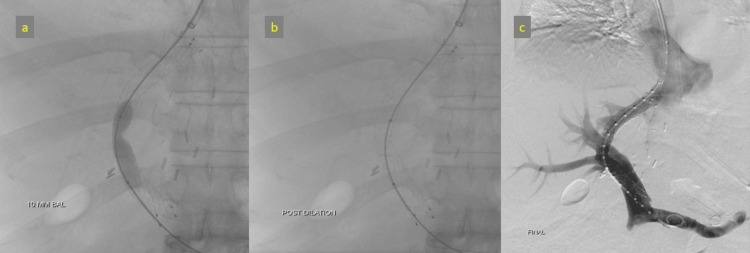
TIPS procedure (a) Shunt track was created across the right hepatic vein and right branch of the portal vein using the Rösch-Uchida set (Cook Medical, Bloomington, USA). (b) The parenchymal tract was dilated, and a 10 mm x 10 cm stent (Niti-S, Taewoong Medical, Gimpo-si, South Korea) was successfully placed. (c) Post-stenting portogram showing good flow through the shunt. TIPS: Transjugular intrahepatic portosystemic shunt

A TIPS procedure was performed via a transjugular approach, with access obtained through the right jugular vein (Figure 12). Under fluoroscopic guidance, a shunt was created from the right hepatic vein to the right portal vein using the Rösch-Uchida set (Cook Medical, Bloomington, USA). The created parenchymal tract was then dilated and stented with a 10 mm x 10 cm endoprosthesis (Niti-S, Taewoong Medical, Gimpo-si, South Korea).

Outcome

Post-TIPS portography confirmed patency and high flow through the shunt, which effectively decompressed the portal system. Ascites resolved, and the patient no longer required paracentesis. This intervention successfully managed portal hypertension, demonstrating TIPS as a critical tool in post-LDLT patients with vascular complications.

Case 4: Portal vein anastomotic stenosis in a post-liver transplant recipient

Patient History

**Figure 13:**
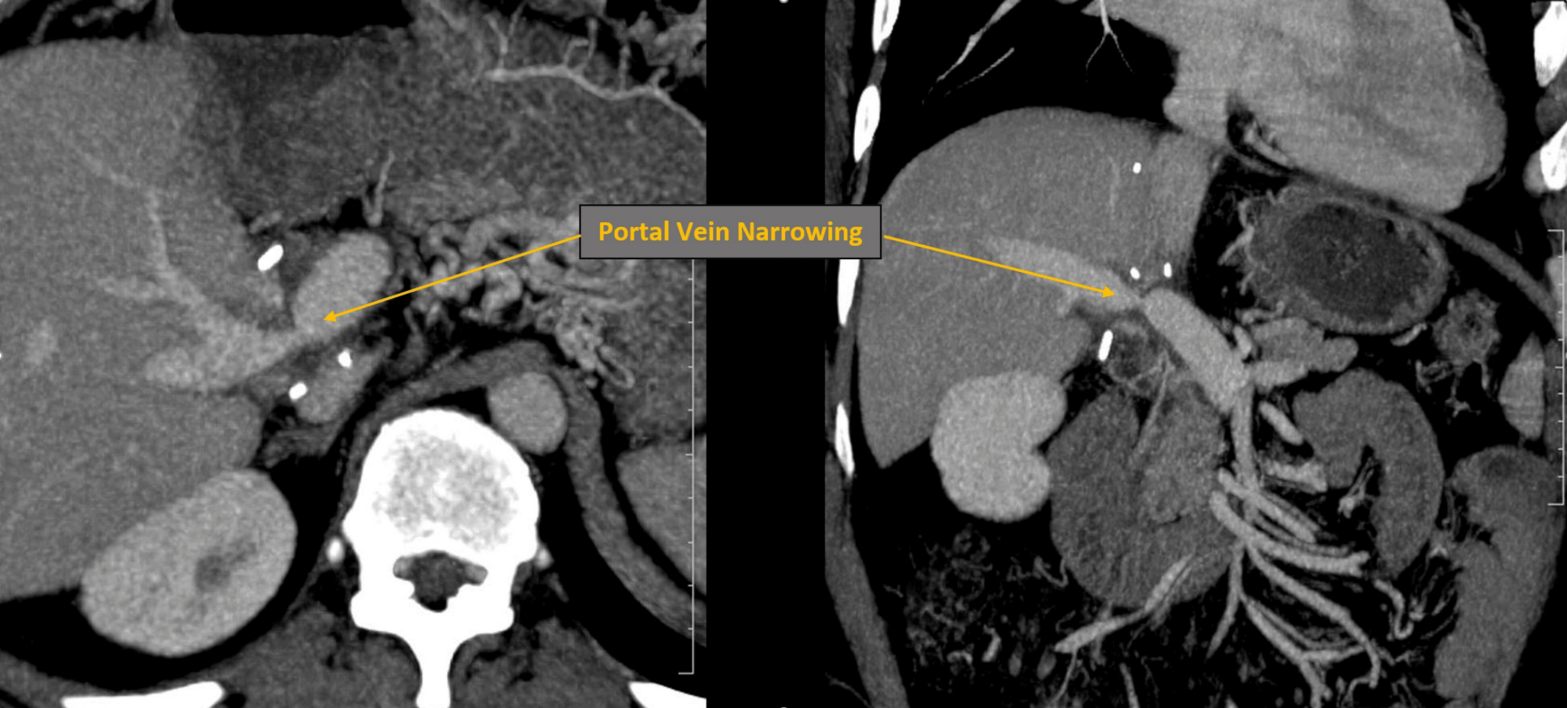
Axial and coronal post-contrast CT images showing portal vein anastomotic stenosis CT: Computed tomography

A 55-year-old male, post-liver transplantation, presented with clinical signs suggestive of portal vein stenosis. Imaging studies, including CT and ultrasound, revealed narrowing at the portal vein anastomotic site. CT imaging showed the maximum luminal diameter at the anastomotic site measuring 4 mm (Figure 13). Ultrasound demonstrated narrowing at the anastomotic site with a post-narrowing dilated segment showing aliasing on colour Doppler.

Intervention

**Figure 14:**
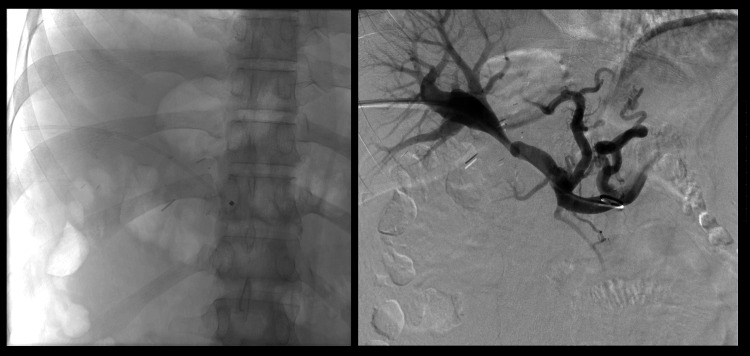
Percutaneous approach portogram showing narrowing of the right portal vein

A portography angioplasty and stenting procedure was performed. The right portal vein was accessed via a percutaneous posterior intercostal route, and a 7F sheath (Cook Medical, Bloomington, USA) was placed. The stenotic anastomotic site was crossed, and pressure measurements showed a gradient of 15 mmHg. Angiography revealed prominent coronary veins and a shelf-like thrombus in the superior mesenteric vein near the main portal vein (Figure 14).

**Figure 15:**
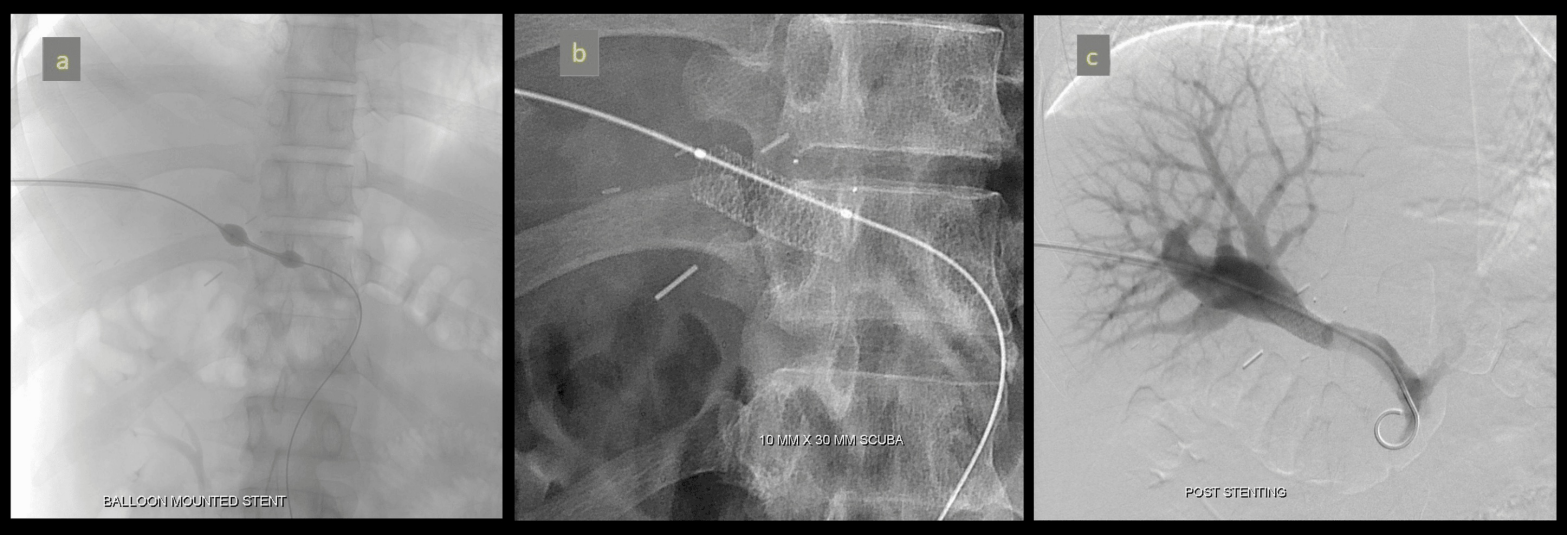
Angiographic image of portal vein stenting (a,b) and good flow across the stent post-stenting (c)

A Kumpe catheter (Cook Medical, Bloomington, USA) was advanced into the superior mesenteric vein near the thrombus, and 3.5 mg of Actilyse was infused over one hour. However, the check angiogram showed no significant change in the size of the clot. Subsequently, a 10 mm x 3 cm balloon-expandable stent (Scuba, Invatec, Italy) was deployed across the stenotic segment. Repeat pressure measurements showed no gradient, with pressures reading 5 mmHg across the portal vein. Post-stenting portogram demonstrated successful filling of the main portal vein and its liver branches, with no further filling of collateral veins (Figure 15).

Outcome

**Figure 16:**
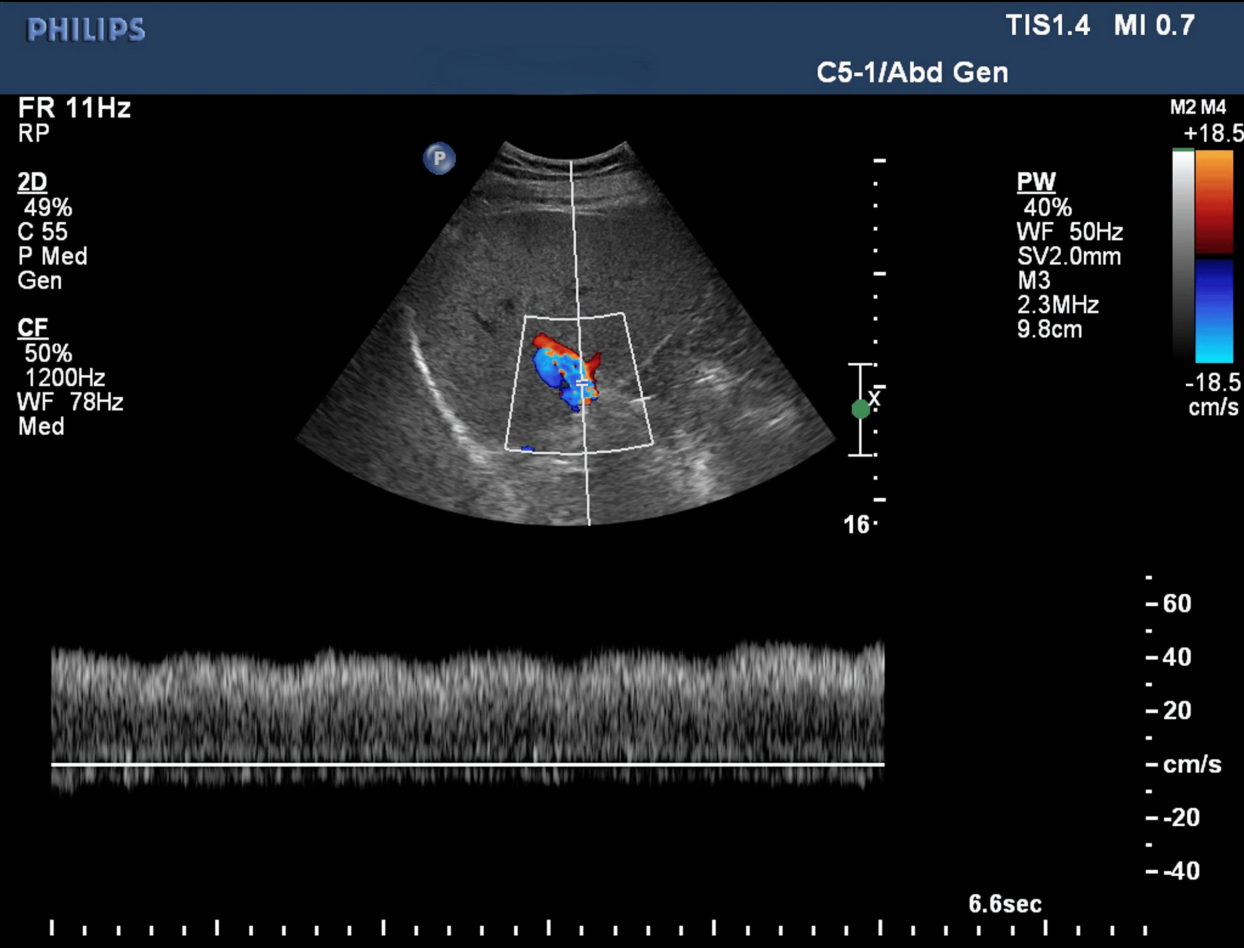
Post-stenting follow-up ultrasound Doppler image showing good flow in portal vein across the stent

Follow-up ultrasound showed good flow across the stent with normalized portal vein velocities, confirming the success of the procedure (Figure 16). The intervention restored portal vein patency and improved hemodynamics, successfully resolving the anastomotic stenosis. The patient has been following up without any major issues for the past 13 years.

## Discussion

LDLT offers an important therapeutic option for patients with end-stage liver disease, expanding access to life-saving procedures. However, post-transplant complications, particularly those involving the biliary and vascular systems, remain a significant cause of morbidity and graft loss. The management of such complications often requires timely and effective interventions to prevent further deterioration. In this context, minimally invasive, image-guided techniques have emerged as critical tools in managing complex post-LDLT complications, offering a safer and more effective alternative to high-risk surgical re-exploration. This case series highlights the role of PTBD, hepatic venoplasty with stenting, and TIPS placement in resolving critical vascular and biliary issues following LDLT.

Biliary complications and PTBD

**Figure 17:**
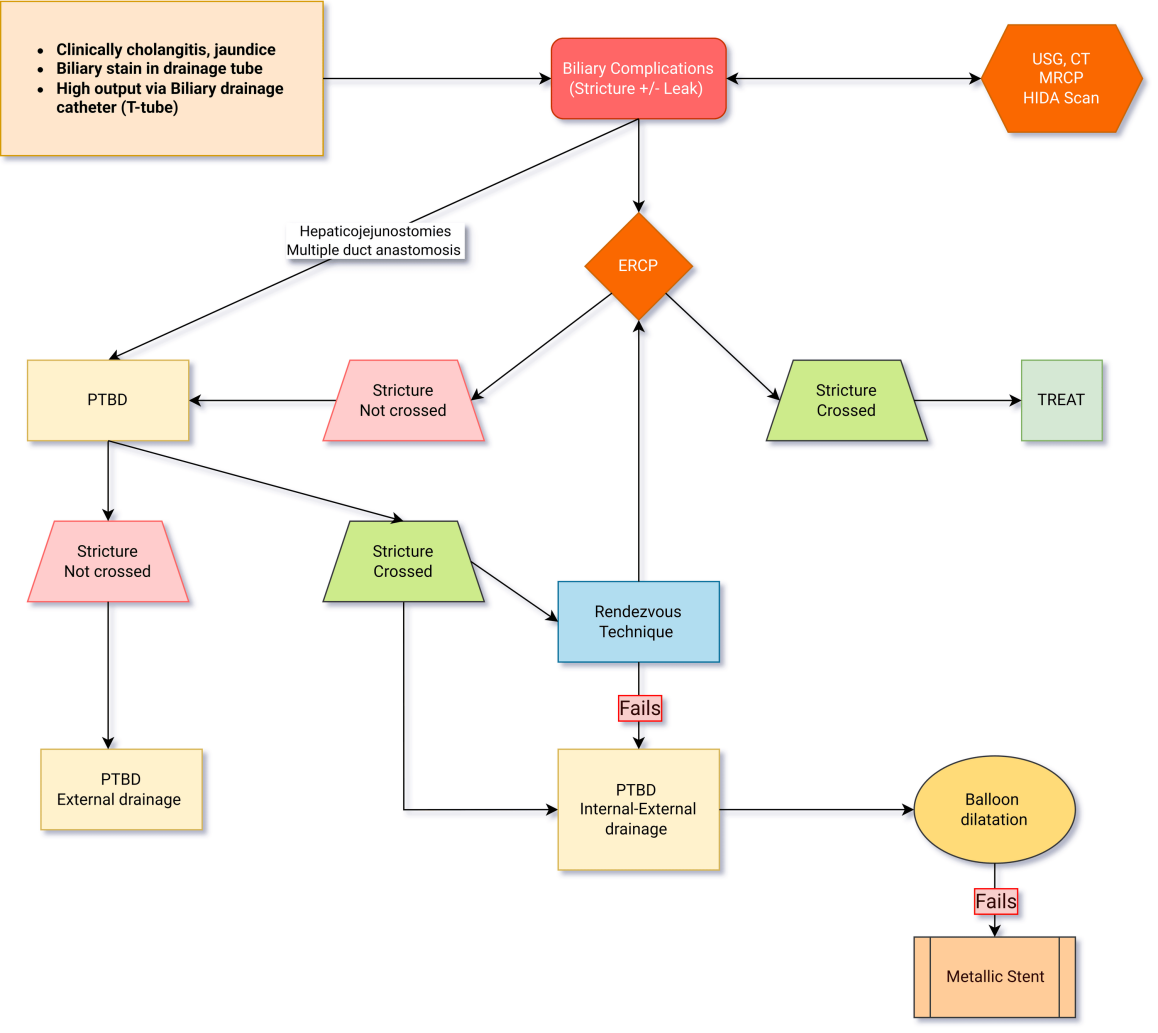
Overview of percutaneous transhepatic approach for biliary complications Percutaneous biliary techniques are effective treatment options with good outcomes in LDLT patients with biliary complications and failed ERCP. Percutaneous techniques have a definite complementary role to ERCP. ERCP: Endoscopic retrograde cholangiopancreatography; LDLT: Living-donor liver transplantation; USG: Ultrasound; CT: Computed tomography; HIDA: Hepatobiliary iminodiacetic acid; MRCP: Magnetic resonance cholangiopancreatography; PTBD: Percutaneous transhepatic biliary drainage Figure is adapted from Kulkarni et al. [2], with permission obtained for its use.

Biliary complications, including anastomotic strictures and leaks, are a well-known challenge in LDLT, affecting up to 30% of the recipients. While ERCP is often the first-line treatment, it may fail due to complex stricture morphology, anatomical variations, or difficulties with access in pediatric patients. In these cases, PTBD with balloon cholangioplasty offers an excellent alternative. Figure 17 depicts an overview of the percutaneous transhepatic approach for biliary complications.

In the first case of this series, a three-year-old pediatric recipient of LDLT presented with biliary anastomotic strictures at the hepatic hilar region. After unsuccessful attempts at ERCP, PTBD was successfully employed to achieve biliary decompression and stricture dilation. PTBD is particularly advantageous when ERCP fails, as it provides direct access to the biliary system, allowing for precise management of strictures with minimal risk of further complications. The ability to use fluoroscopic guidance during balloon cholangioplasty ensures the optimal expansion of the stenotic ducts, improving bile flow and promoting healing without the need for invasive surgical revision. As seen in our case, PTBD led to the normalization of liver function and resolution of hepatic hilar collections, demonstrating the procedure's utility in complex biliary management. These findings align with previous studies that highlight PTBD as a highly effective technique for managing post-transplant biliary strictures, with a low complication rate.

Risk of Restenosis

Restenosis is a known risk following balloon cholangioplasty, particularly in cases of biliary anastomotic strictures after LDLT. While balloon cholangioplasty is an effective and minimally invasive treatment for biliary strictures, there is a possibility of recurrent narrowing of the bile duct at the site of the intervention, which can lead to the need for repeat procedures. Restenosis occurs due to the fibrotic nature of the biliary strictures, and the mechanical stress exerted by the balloon during dilation can lead to intimal hyperplasia or scarring, causing the duct to narrow again. Several factors can influence the risk of restenosis, including the complexity of the stricture, status of the hepatic artery and biliary perfusion, the size of the balloon used, and the patient’s underlying liver condition.

To minimize the risk of restenosis, some studies recommend the use of stent placement after balloon dilation, particularly in cases where the stricture is severe or recurrent. Self-expandable metallic stents (SEMS) are particularly effective in such scenarios, as they provide durable long-term patency and reduce the need for repeated interventions like balloon dilations or catheter repositioning.

**Figure 18:**
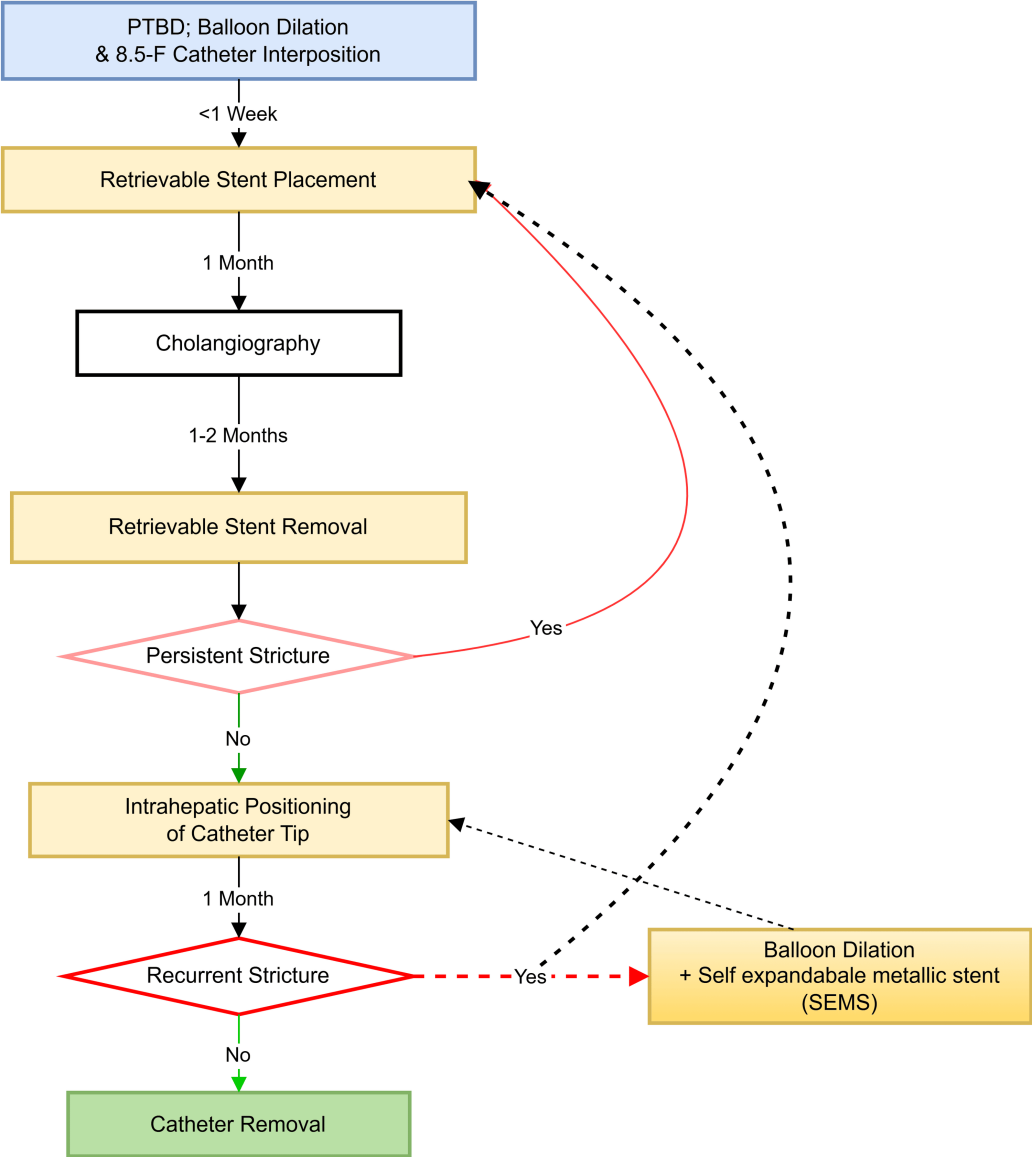
Approach to restenosis In restenosis, SEMS provide durable long-term patency and reduce the need for repeated interventions. SEMS: Self-expandable metallic stents; PTBD: Percutaneous transhepatic biliary drainage Figure credits: Author Bribin Bright

However, in our institution (Figure 18), stenting is considered a last resort due to the potential complications associated with biliary stenting, such as stent occlusion, migration, fracture, and tissue ingrowth, which may lead to recurrent strictures or bile duct obstruction. While SEMS can be beneficial in selected cases, especially for refractory or complex strictures, they do not eliminate the risk of restenosis or other long-term complications. Additionally, SEMS carry challenges such as higher costs and risks of migration, occlusion due to sludge or stones, and tissue ingrowth, particularly with uncovered or partially covered stents. Despite these limitations, SEMS remains an essential option for managing complex biliary strictures, offering long-term relief and reducing the need for repeated procedures. Their use should be individualized, balancing their benefits and potential risks, with close follow-up to monitor outcomes and address complications effectively.

Vascular complications and percutaneous venoplasty

Vascular complications, particularly HVOO caused by hepatic vein or IVC stenosis, are known to contribute to graft dysfunction, ascites, and other hemodynamic abnormalities. Open surgical repair or revision can be technically challenging and carries significant risks, including bleeding, infection, and increased mortality. Percutaneous venoplasty and stenting, however, provide a minimally invasive alternative with excellent outcomes.

In the second case, an adult recipient with HVOO secondary to IVC stenosis and hepatic vein occlusions was successfully treated using percutaneous venoplasty with stent placement. The deployment of a balloon-expandable stent restored patency to the hepatic veins, resolving the outflow obstruction. The use of venography for precise identification of the stenotic segments, combined with balloon dilation and stenting, offers an effective method for managing vascular complications that avoids the high morbidity associated with open surgery. Follow-up venography confirmed the resolution of the obstruction, improvement in hemodynamics, and relief of symptoms such as ascites, highlighting the advantages of this technique in treating post-LDLT vascular issues. These findings are consistent with the literature that supports the long-term success of percutaneous venoplasty and stenting in the management of HVOO, especially in patients with altered anatomy post-transplantation.

Additionally, in the case of portal vein anastomotic stenosis, percutaneous intervention with balloon-expandable stent placement successfully restored portal vein patency. After thrombolytic treatment for a superior mesenteric vein thrombus, follow-up imaging confirmed the resolution of the stenosis, with restored flow and no significant gradient, underscoring the effectiveness of stenting in managing post-transplant portal vein complications.

TIPS for portal hypertension and refractory ascites

Portal hypertension and refractory ascites are frequent sequelae of liver transplant, often resulting from graft congestion or hepatic vein stenosis. In these cases, the standard treatment of paracentesis is temporary, and repeat procedures are often needed to control symptoms. The TIPS procedure, a well-established technique for portal hypertension, has proven to be a valuable option in managing these complications by decompressing the portal system and reducing portal pressure.

In our third case, TIPS was successfully performed in a patient with refractory ascites secondary to portal hypertension following LDLT. The creation of a shunt between the right hepatic vein and the portal vein restored normal portal pressure, alleviating the need for repeated paracentesis and significantly improving the patient's quality of life. The procedural success, along with the resolution of ascites, underscores the utility of TIPS in managing post-transplant portal hypertension and ascites. Numerous studies support the safety and efficacy of TIPS in this population, demonstrating its ability to improve clinical outcomes and reduce the frequency of invasive procedures like paracentesis. 

Risk of hepatic encephalopathy following TIPS: Prevention and the role of LFTs

**Figure 19:**
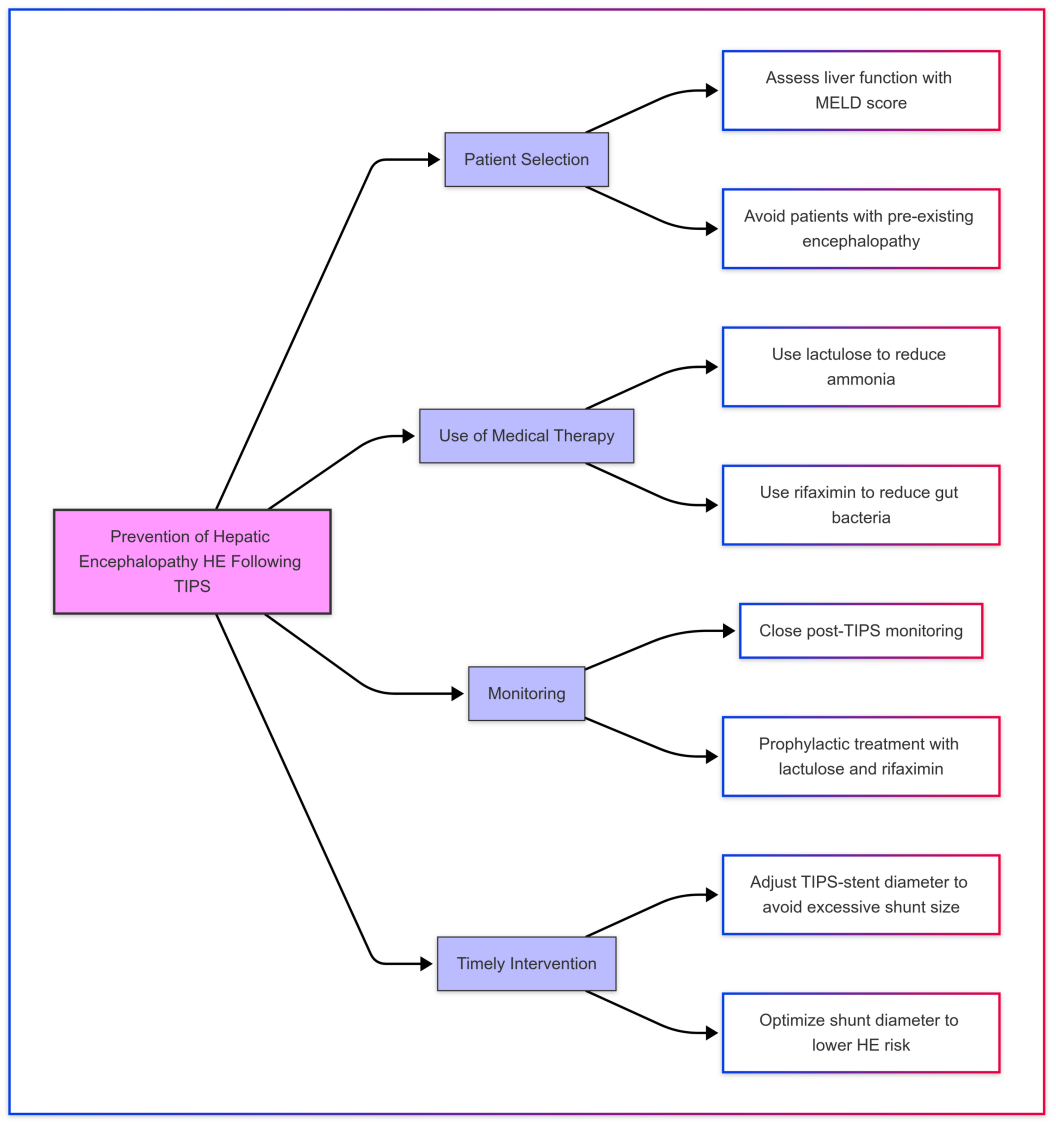
Key strategies for prevention of hepatic encephalopathy following TIPS TIPS: Transjugular intrahepatic portosystemic shunt; MELD: Model for End-Stage Liver Disease; HE: Hepatic encephalopathy Figure credits: Author Bribin Bright

Hepatic encephalopathy is a significant concern following a TIPS procedure, which is typically performed to treat complications of portal hypertension, such as variceal bleeding or refractory ascites. The risk of developing hepatic encephalopathy after TIPS is a well-known complication due to the diversion of blood from the portal circulation to the systemic circulation. Preventing hepatic encephalopathy following TIPS involves careful patient selection, early identification of risk factors, and appropriate medical management, as described in Figure 19. For this, we work as a multidisciplinary team with our hepatologist and gastroenterologist. Assessing liver function before performing TIPS is essential. Using scoring systems such as the Model for End-Stage Liver Disease (MELD) score can help identify patients at higher risk. Medications like lactulose, which reduces ammonia levels in the blood, and rifaximin, an antibiotic that reduces gut bacteria responsible for producing ammonia, are commonly used to prevent or treat hepatic encephalopathy following TIPS. Close monitoring of the patient after TIPS is essential. Prophylactic treatment with lactulose and rifaximin may be indicated. The diameter of the shunt can influence the incidence of hepatic encephalopathy. Larger shunts may be associated with a higher risk, and therefore, careful selection of shunt size is critical in reducing complications like hepatic encephalopathy. Adjusting the TIPS-stent diameter to avoid excessive shunt size may lower the risk of encephalopathy. 

Advantages of image-guided interventions

This case series emphasizes the growing role of interventional radiology in post-transplant care. Minimally invasive image-guided interventions offer numerous advantages over traditional surgical approaches, including lower morbidity, reduced hospital stays, and faster recovery times. Furthermore, these procedures are particularly valuable in managing complex complications in patients with altered anatomy, where conventional surgical techniques may be more difficult or risky. PTBD, venoplasty, and TIPS not only offer effective treatment options but also allow for real-time monitoring and precision, minimizing the risk of injury to surrounding structures.

The four cases illustrate the versatility and efficacy of minimally invasive, image-guided interventions in the management of biliary and vascular complications following LDLT. Each case presented a unique clinical challenge, which was effectively managed through percutaneous techniques, thus avoiding the high-risk alternative of surgical re-exploration.

The success of these interventions is also supported by the growing body of evidence, which indicates that these techniques, when performed by skilled interventional radiologists, can achieve outcomes comparable to or even better than surgical alternatives. The ability to intervene early and avoid surgical re-exploration can significantly reduce the burden on healthcare systems while improving patient survival rates and quality of life.

## Conclusions

In conclusion, image-guided interventions, such as PTBD, venoplasty with stenting, and TIPS, are pivotal in managing biliary and vascular complications following LDLT. These minimally invasive techniques offer effective solutions with reduced procedural risk and morbidity, providing an alternative to high-risk surgical re-exploration. By improving graft survival and patient outcomes, interventional radiology plays a crucial role in the multidisciplinary management of post-transplant complications, ultimately enhancing the longevity of the graft and the patient's quality of life. The idea is not to live forever but maybe to help another live a little longer.
